# Synaptic Plasticity in Neural Networks Needs Homeostasis with a Fast Rate Detector

**DOI:** 10.1371/journal.pcbi.1003330

**Published:** 2013-11-14

**Authors:** Friedemann Zenke, Guillaume Hennequin, Wulfram Gerstner

**Affiliations:** 1 School of Computer and Communication Sciences and School of Life Sciences, Brain Mind Institute, Ecole polytechnique fédérale de Lausanne, Lausanne, Switzerland; 2 Computational and Biological Learning Laboratory, Department of Engineering, University of Cambridge, Cambridge, United Kingdom; Research Center Jülich, Germany

## Abstract

Hebbian changes of excitatory synapses are driven by and further enhance correlations between pre- and postsynaptic activities. Hence, Hebbian plasticity forms a positive feedback loop that can lead to instability in simulated neural networks. To keep activity at healthy, low levels, plasticity must therefore incorporate homeostatic control mechanisms. We find in numerical simulations of recurrent networks with a realistic triplet-based spike-timing-dependent plasticity rule (triplet STDP) that homeostasis has to detect rate changes on a timescale of seconds to minutes to keep the activity stable. We confirm this result in a generic mean-field formulation of network activity and homeostatic plasticity. Our results strongly suggest the existence of a homeostatic regulatory mechanism that reacts to firing rate changes on the order of seconds to minutes.

## Author Summary

Learning and memory in the brain are thought to be mediated through Hebbian plasticity. When a group of neurons is repetitively active together, their connections get strengthened. This can cause co-activation even in the absence of the stimulus that triggered the change. To avoid run-away behavior it is important to prevent neurons from forming excessively strong connections. This is achieved by regulatory homeostatic mechanisms that constrain the overall activity. Here we study the stability of background activity in a recurrent network model with a plausible Hebbian learning rule and homeostasis. We find that the activity in our model is unstable unless homeostasis reacts to rate changes on a timescale of minutes or faster. Since this timescale is incompatible with most known forms of homeostasis, this implies the existence of a previously unknown, rapid homeostatic regulatory mechanism capable of either gating the rate of plasticity, or affecting synaptic efficacies otherwise on a short timescale.

## Introduction

The awake cortex is constantly active, even in the absence of external inputs. This baseline activity, commonly referred to as the “background state”, is characterized by low synchrony at the population level and highly irregular firing of single neurons. While the direct implications of the background state are presently unknown, several neurological disorders such as Parkinson's disease, epilepsy or schizophrenia have been linked to various disruptions thereof [1]–[5]. Theoretically, the background state is currently understood as the asynchronous and irregular (AI) firing regime resulting from a dynamic balance of excitation and inhibition in recurrent neural networks [6]–[9]. Balanced networks exhibit low activity and small mean pairwise correlations [7], [9]. However, even small changes in the amount of excitation can disrupt the background state [7], [10]. Changes in excitation can arise from Hebbian plasticity of excitatory synapses: Subsets of jointly active neurons form strong connections with each other which is thought to be the neural substrate of memory [11]. However, Hebbian plasticity has the unwanted side effect of further increasing the excitatory synaptic drive into cells that are already active. The emergent positive feedback loop renders this form of plasticity unstable and makes it hard to reconcile with the stability of the background state [12].

To stabilize neuronal activity, homeostatic control mechanisms have been proposed theoretically [13]–[19] and various forms have indeed been found experimentally [20]–[22]. The term homeostasis comprises any compensatory mechanism that stabilizes neural firing rates in the face of plasticity induced changes. This includes compensatory changes in the overall synaptic drive (e.g. synaptic scaling [21]), the neuronal excitability (intrinsic plasticity [23]) or changes to the plasticity rules themselves (i.e. metaplasticity [20]). Common to all experimentally found homeostatic mechanisms is their relatively slow response compared to plasticity. While synaptic weights can change on the timescale of seconds to minutes [24]–[26], noticeable changes caused by homeostasis generally take hours or even days [21], [27]–[29]. This is thought to be crucial since it allows neurons to detect their average firing rate by integrating over long times. While fluctuations on short timescales cause Hebbian learning and alter synapses in a specific way to store information, at longer timescales homeostasis causes non-specific changes to maintain stability [23]. The required homeostatic rate detector acts as a low-pass filter and therefore induces a time lag between the rate estimate and the true value of neuronal activity. As a result, homeostatic responses based on this detector become inert to sudden changes. The longer the filter time constant is, the more sluggish the homeostatic response becomes.

Here we formalize the link between stability of network activity and the timescales involved in homeostasis in the presence of Hebbian plasticity. We first study the stability of the background state during long episodes of ongoing plasticity in direct numerical simulations of large balanced networks with a metaplastic triplet STDP rule [30] in which the timescale of homeostasis is equal to the one of the rate detector. This allows us to determine the critical timescale beyond which stability is lost. In a second step we reduce the system to a generic two-dimensional mean-field model amenable to analytical considerations. Both the numerical and the analytical approach show that homeostasis has to react to rate changes on a timescale of seconds to minutes. We then show analytically and in simulations that these stability requirements are not specific to metaplastic triplet STDP, but generalize to the case of triplet STDP in conjunction with synaptic scaling.

In summary we show that the stability of the background state requires the ratio between the timescales of homeostasis and plasticity to be smaller than a critical value 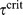 which is determined by the network properties. For realistic network and plasticity parameters this requires the homeostatic timescale to be short, meaning that homeostasis has to react quickly to changes in the neuronal firing rate (on the order of seconds to minutes). Our results suggest that plasticity must either be gated rapidly by a third factor, or be accompanied by a yet unknown homeostatic control mechanism that reacts on a short timescale.

## Results

In the following we first discuss our results obtained from simulating spiking neural networks in the balanced state with a Hebbian learning rule subject to a plausible learning rate. In the beginning we focus on a metaplastic mechanism that regulates the amount of synaptic long term depression (LTD) homeostatically. By systematically varying the time constant of the homeostatic rate detector, we find that stability of the background state requires homeostasis to act on a timescale of minutes. We then strive to understand the underlying mechanism of the instability from a generic mean field model, which we use to analytically confirm the critical time constant found in the spiking network simulations. Finally, to explore the generality of this mean field approach, we apply the analysis to two variations of the triplet learning rule. First, we add a slow weight decay to metaplastic triplet STDP and second we switch from homeostatic metaplasticity to synaptic scaling in combination with triplet STDP. In both cases we confirm analytically and in simulations that a fast rate detector is required to assure stability.

### Simulation results

**Figure 1:**
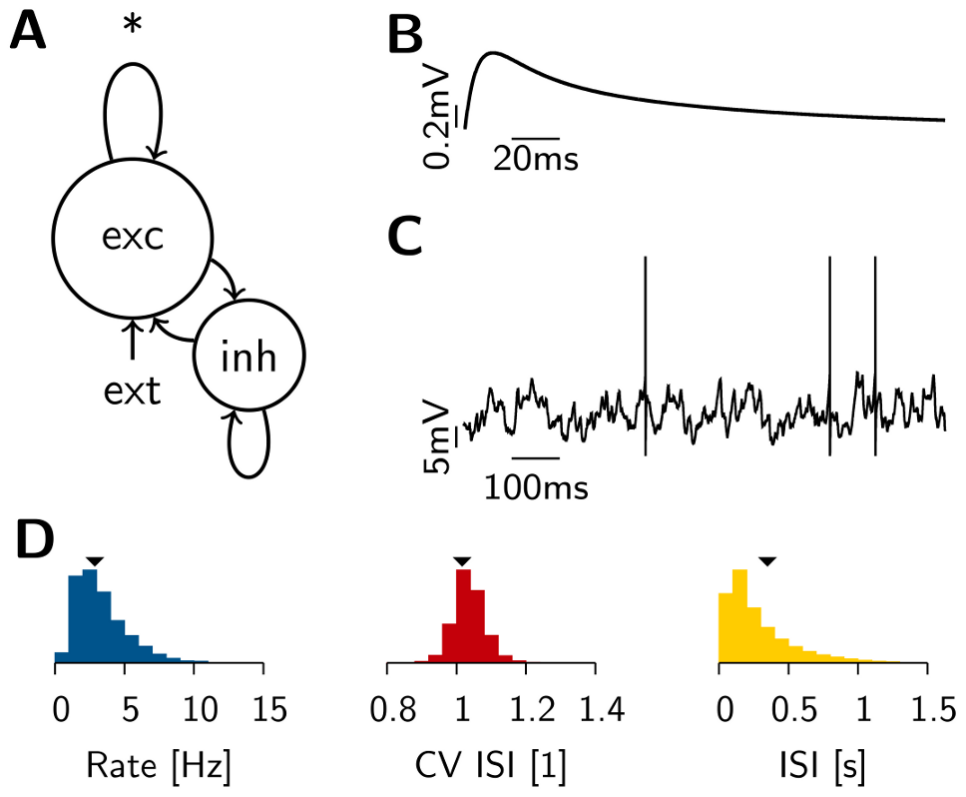
The balanced network model. (**A**) Schematic of the network model. Recurrent synapses in the population of excitatory neurons (*) are subject to the homeostatic triplet STDP rule. (**B**) Typical magnitude and time course of a single excitatory postsynaptic potential from rest. (**C**) Membrane potential trace of a cell during background activity. (**D**) Histogram of single neuron firing rates (blue) and coefficient of variation (CV ISI, red) across neurons as well as the ISI distribution of all neurons (yellow) of the network during background activity. Arrowheads indicate mean values.

To study the stability of the background state in balanced networks with plastic excitatory-to-excitatory (EE) synapses we simulate networks of 25000 randomly connected integrate-and-fire neurons (Figure 1 A). Prior to any synaptic modification by plasticity, we set the network to the balanced state in which membrane potentials exhibit large sub-threshold fluctuations (Figure 1 C), giving rise to irregular activity at low rates (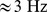) and asynchronous firing at the population level (Figure 1 D). In our model more than 90% of the input to each neuron comes from within the network, thus closely resembling conditions found in cortex [31].

Plasticity of all recurrent EE synapses is modeled as an additive triplet STDP rule (see [30] and Methods) which accurately describes experimental data from visual cortex [26], [30]. In this metaplastic triplet STDP rule the amount of LTD is chosen such that LTP and LTD cancel on average, when the pre- and postsynaptic neurons fire with Poisson statistics at rate 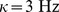. Therefore, under the assumption of low spike-spike correlations and irregular firing, 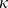 becomes a fixed point of the network dynamics (see [32] and Methods). We begin with a fixed learning rate 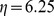, which is chosen as a compromise between biological plausibility and computational feasibility (Methods). To go towards the fixed point, all neurons constantly estimate their firing rate as the moving average 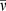 with exponential decay constant 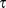, given by
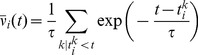
(1)where 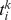 corresponds to the 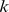-th firing time of neuron 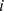 (see also Methods, Eq. (19)). If the rate estimate 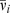 of the postsynaptic neuron 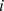 lies above (below) 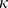, homeostasis increases (decreases) the LTD amplitude. The homeostatic time constant 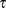 is the only free parameter of our model.

**Figure 2:**
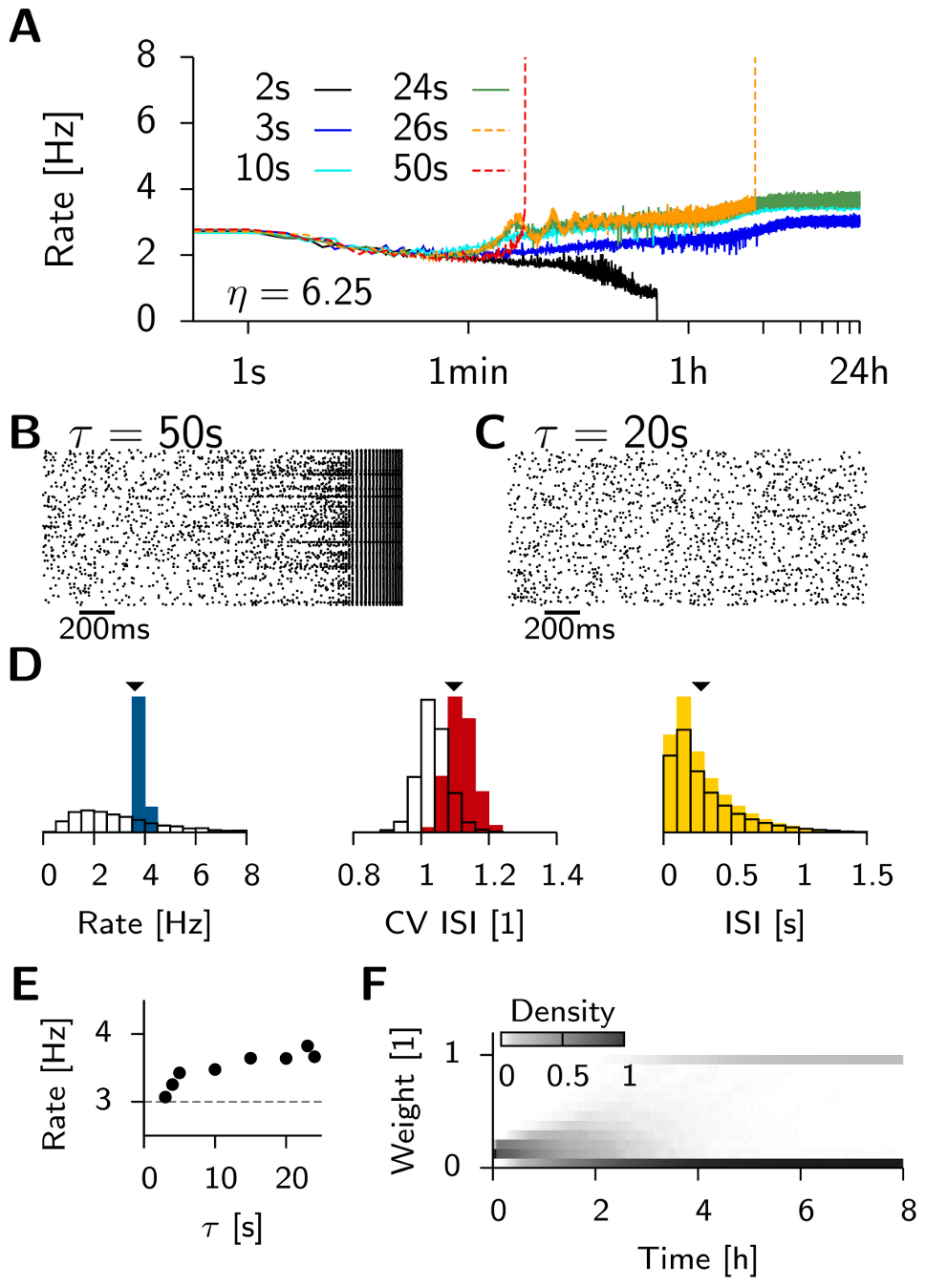
Network stability during ongoing synaptic plasticity depends crucially on the homeostatic time constant. (**A**) Temporal evolution of the average firing rate in the excitatory population for different homeostatic time constants 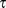. Explosion of firing rate indicated by dashed lines. Curves for 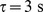 (dark blue), 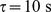 (light blue), and 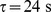 (turquoise) overlap on the interval from 2 h to 24 h indicating stability. With 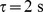 (black) we show one of the cases with very short 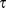 where the activity spontaneously dies. (**B**) Spike raster of 200 randomly selected excitatory neurons. The last two seconds are shown before the network activity destabilizes (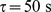). (**C**) For 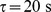, the activity stays asynchronous and irregular even after 24 h hours of simulated time. (**D**) Firing statistics in a stable network (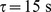) measured after 24 h of simulated time. Histogram of single neuron firing rates (blue) and coefficient of variation (CV ISI, red) across neurons and the ISI distribution of all neurons (yellow). Arrowheads indicate mean values. Black lines represent the corresponding statistics prior to any synaptic modifications (copied from Figure 1). (**E**) Population firing rate for stable simulation runs at 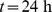 as a function of the homeostatic time constant. The dashed line indicates the target firing rate 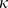. (**F**) Evolution of the synaptic weight distribution during the first 8 hours of synaptic plasticity (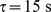).

We then explore systematically how a particular choice of 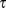 affects the stability of the background state in the network. To allow the moving averages to settle, we run the network for an initial period of duration 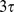, during which synaptic updates are not carried out. After that, plasticity is switched on. To check whether the network dynamics remain stable, simulations are run for 24 h of biological time during which we constantly monitor the evolution of the population firing rate (Figure 2 A). The network is considered unstable if the mean population firing rate either drops to zero or increases above 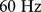 which happens when run-away potentiation occurs (Figure 2 B). By systematically varying the time constant 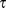 in 1 s steps, we find that for the background state to remain stable (Figure 2 C), 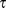 must be shorter than some critical value 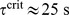. Moreover, we find a sharp transition to instability when 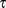 is increased beyond 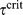. For 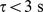 the network has a tendency to fall silent (Figure 2 A, black line).

During stable simulation runs (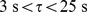), some synapses grow from their initial value 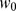 up to the maximum allowed value 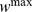, while the rest of the synapses decay to zero. The resulting bimodal distribution of synaptic efficacies (Figure 2 F) remains stable until the end of the run. This is a known phenomenon for purely additive learning rules [33], [34] and we will see later that unimodal weight distributions arise by the inclusion of a weight decay or by choosing synaptic scaling as the homeostatic mechanism [35].

Despite the qualitative change in the weight distribution, the inter-spike-interval (ISI) distribution remains largely unaffected, while the coefficient of variation of the ISI distribution (CV ISI) is shifted to slightly higher values (Figure 2 D). However, we noted that the single-neuron average firing rates, which are widely spread out initially, are at the end clustered slightly above the homeostatic target rate of (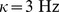) with a weak dependence on the actual value of 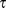 (Figure 2 E). This behavior is characteristic for homeostatic firing rate control in single cells.

We conclude that metaplastic triplet STDP with a homeostatic mechanism as presented here can lead to stable dynamics in models of balanced networks exhibiting asynchronous irregular background activity. However, the timescale 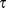 of the homeostatic mechanism critically determines stability. It has to be on the order of seconds to minutes and therefore comparable to the timescale of plasticity itself (here 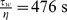). This finding is in contrast to most known homeostatic mechanisms that have experimentally been found to act on effective timescales of hours or days [20], [29], [36], [37].

### Mean field model

**Figure 3:**
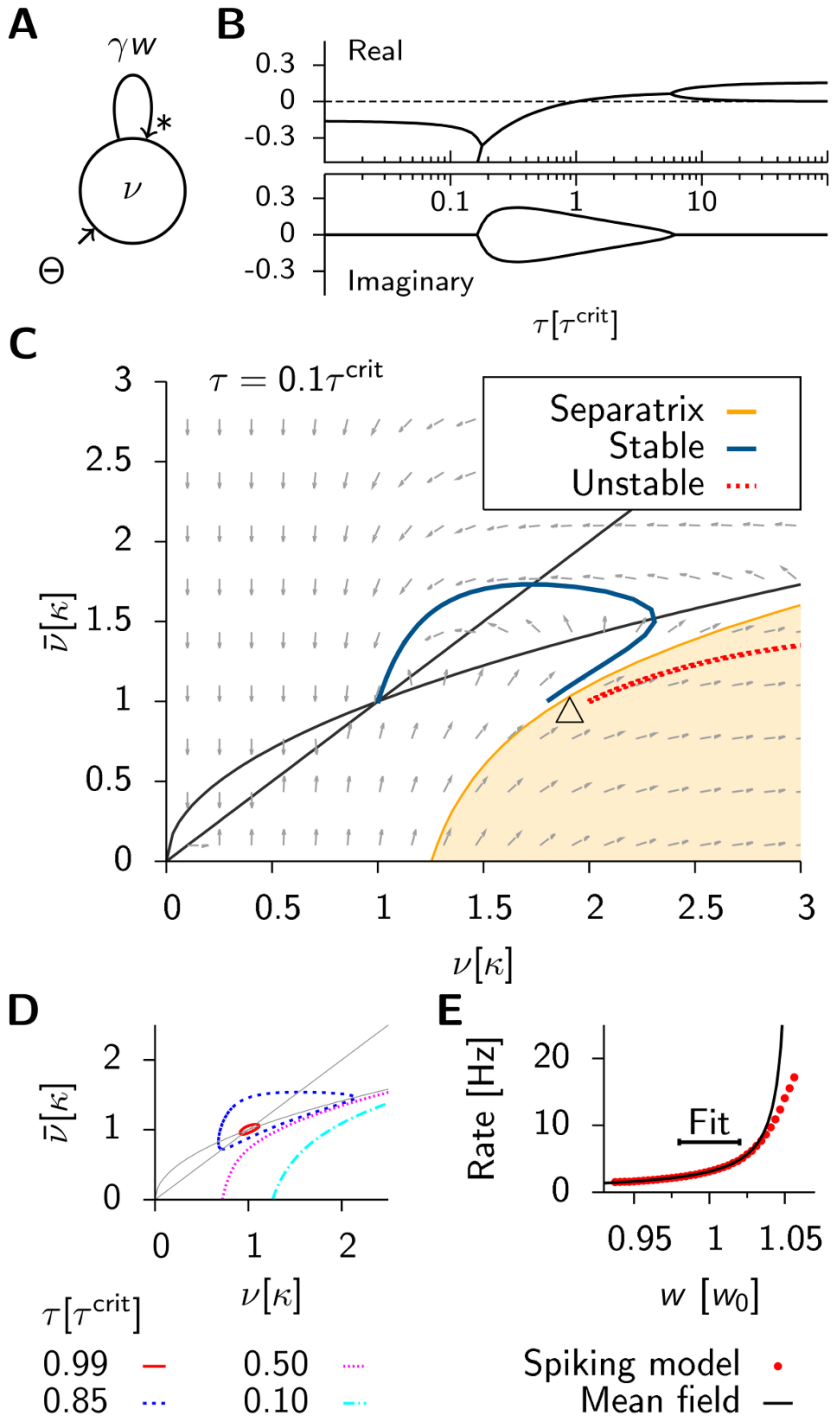
Mean field theory predicts the stability of background activity. (**A**) Schematic of the mean field model. Plastic synapses are indicated by *. (**B**) Eigenvalues of the Jacobian evaluated at the non-trivial fixed point 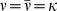. (**C**) Phase portrait for 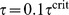, a choice where background activity is stable. Nullclines are drawn in black. Arrows indicate the direction of the flow. Two prototypical trajectories starting close to 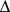 are shown. Blue line: Typical example of a solution that returns to the stable fixed point. Solutions starting in the shaded area, such as the red line, diverge to infinity. (**D**) The separatrix for four different values of 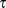. (**E**) Population firing rate of the spiking network model (simulations: red dots) for different values of weight 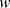 for connections from excitatory to excitatory neurons. Black line: Least-square fit of Eq. (3) on the interval 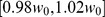 as indicated by the black bar. Extracted parameters are 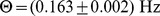 and 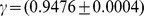 (cf. Eq. (3)).

To understand why the critical time constant 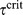 above which homeostasis cannot control plasticity is so short, we here analyze the stability of the background state in a mean field model. In line with the spiking network model we consider a single population of neurons that fires with the mean population firing rate 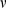 (Figure 3 A). To find an analytic expression that characterizes the response of the background activity to changes in the recurrent weights 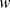 around the initial value 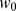, we begin with a linear neuron model
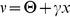
(2)with the offset 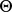 and the slope parameter 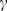. Since we are interested in weight changes around the initial value 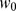, the natural choice for 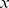 would be 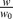. However, here we set 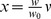 to take into account the recurrent feed-back. This choice makes 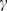 dimensionless while 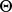 is measured in units of Hz. Because weights evolve slowly, while population dynamics are fast we can solve for 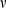 and obtain the self-consistent solution
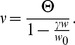
(3)


As we will show later, a better qualitative fit to the spiking model can be achieved with this heuristic, which will facilitate finding the right parameters 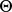 and 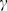.

To introduce plasticity into the mean field model, we use the corresponding rate-based plasticity rule
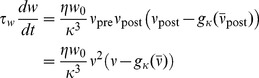
(4)which can be directly derived from the triplet STDP rule [30] and also can be interpreted as a BCM model [15], [30], [38]. Here, 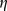 is the relative learning rate and 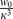 sets the scale of the system. The second equality in Eq. (4) follows because in the recurrent model pre- and postsynaptic rates are the same (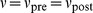 and 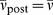). The function 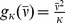 scales the strength of LTD relative to LTP just as in the spiking case (cf. Methods, Eq. (18)). In the mean field model, the rate detector 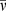 (Eq. (1)) becomes the low pass filtered version of the population firing rate
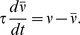
(5)


To link the network dynamics with synaptic plasticity we take the derivative of Eq. (3), 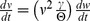 and combine it with Eq. (4) to arrive at
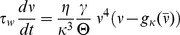
(6)which describes the temporal evolution of the mean firing rate as governed by synaptic plasticity. Taken together, equations (5) and (6) define a two-dimensional dynamical system with two fixed points. One lies at 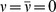 and represents the quiescent network. The remaining non-trivial fixed point is 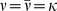, which we interpret as the network in its background state.

Given these choices, we now ask whether this fixed point can be linearly stable (Methods) and find that the stability of the background state requires
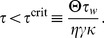
(7)


For 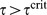 infinitesimal excursions from the fixed point diverge, which corresponds to run-away potentiation in this model. We note that 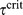 crucially depends on the parameters 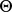, 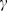, 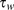, 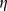 and the target rate 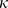. However, we can rescale the system to natural units, by expressing firing rates in units of 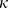 and time in units of 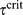, and plot the eigenvalues as a function of 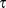 (Figure 3 B). The fact that the fixed point of background activity loses stability for too large values of 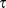 is in good qualitative agreement with what we observe in the spiking model. One should further note that Eq. (7) is independent of the power of 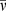 appearing in 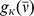, as long as the fixed point of background activity exists (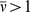) and under the condition that at criticality the imaginary parts of the eigenvalues are always non-vanishing (see Methods). This indicates the presence of oscillations which are indeed observed in the spiking network (cf. Figure 2 A, 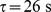). The fact that the network falls silent for very small values of 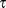 (e.g. 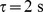 in Figure 2 A) is not captured by the mean field model.

We can make further use of the mean field model to qualitatively understand the behavior of the system far from equilibrium. Figure 3 C shows the phase plane of a network with a stable fixed point (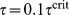). When the system is driven away from it, and perturbations are small, the dynamics converge back towards the fixed point. However, when excursions become too large, the network activity diverges (compare Figure 3 C, dotted solution) since the fixed point of background activity is only locally stable. A numerical analysis shows that the basin of attraction is small when 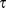 approaches 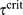 from below (Figure 3 D). Hence the system is very sensitive to perturbations which easily lead to run-away potentiation. Although we expect the basin of attraction of the mean-field model and the spiking model only to be comparably where Eq. (3) describes the firing rates of the spiking network accurately we can assume that for robust stability 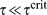 has to be satisfied.

### Model comparison

To be able to make more quantitative predictions for the spiking network we have to choose values for the parameters on the right hand side of Eq. (7). These are the effective timescale of plasticity 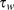 on the one hand, and 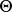 and 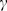, which characterize the network dynamics, on the other hand. We will now show that the latter can be determined from the static network model, which is independent of plasticity. Note that the parameters 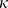 and 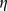 in our mean field model are shared with the spiking model which we will use to quantitatively compare the two.

First, we relate the variables 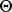 and 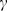 to the response of the spiking network when all its EE synapses are modified. Since this is not feasible analytically, we extract the response numerically by systematically varying the EE weights around the initial state with 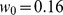. While doing so, plasticity is disabled and we record the steady state population rate of the network (Figure 3 E). We then minimize the mean square error for Eq. (3) over a small interval 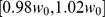 and determine the following values: 
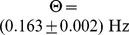
 and 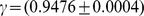. For the stability analysis only the derivative of Eq. (3) at 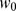 matters. However, it is worth noting that the response of the balanced network is well captured by Eq. (3) over a much wider range than the one used for the fit. This behavior is an expected consequence of the balanced state, which is known to linearize network responses [6], [39]. Our approximation by a linear rate model breaks down for higher rates since it does not incorporate refractory effects.

Second, under the assumption of independent and irregular firing in the background state, the plasticity time constant 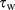 is fully determined by the target rate 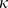 and known parameters of the triplet STDP model (see Methods and [30]). For 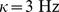 we find 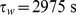.

**Figure 4:**
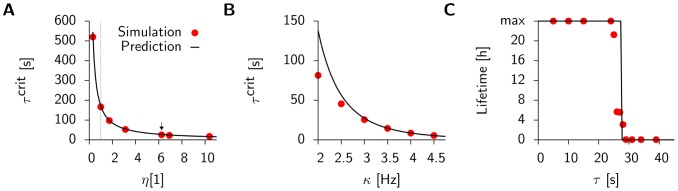
The mean field predictions agree with results from direct simulation of the spiking network. (**A**) Solid line: 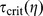 as a function of the learning rate 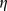 (cf. Eq. (7)), with simulation data (red points) for 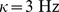. The arrow indicates the value used throughout the rest of this figure (the dotted line corresponds to the learning rate 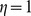 as used in Figure S1). (**B**) Same as before but as a function of 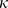 for 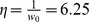 fixed. (**C**) Lifetime values for the spiking network (red points) with a scaled step function as predicted by mean field theory (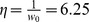 and 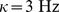). All error bars are smaller than the data points.

Using these results together with Eq. (7) we predict the critical timescale of homeostasis for different values of 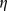 and 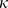 and compare it to the results that we obtain as before from direct simulations of the spiking network. Figure 4 A shows that the dependence of 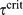 on the learning rate 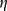 is remarkably well captured by the mean field model. The fourth power dependence on the background firing rate 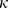 is described well for 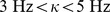 (Figure 4 B), but the theory fails for smaller values, where we start to observe synchronous events in the population activity, which introduce correlations that are not taken into account in the mean field approach. In Figure 4 C we plot the typical lifetimes (i.e. the time when the spiking simulations are stopped, because they either show run-away potentiation or the maximum simulated time 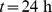 is reached) as a function of 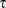. The figure illustrates nicely that the critical time constant 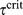 coincides with the sharp transition in lifetimes observed in the spiking network.

When running additional simulations with smaller learning rates (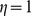 as opposed to 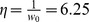) we observe that the network destabilizes occasionally for values of 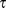 smaller than 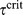, but only after 22 h of activity (see Figure S1). We find, however, that this “late” instability can be avoided by either initializing the EE weights with a weight matrix obtained from a stable run (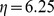 at 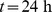) or by reducing the maximally allowed synaptic weight (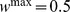). Since these changes do not affect the “early” instability (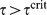), the “late” instability seems to have a different origin and might be linked to the spontaneous emergence of structure in the network.

Here we focus on the “early” instability which is seen in all simulations that do not respect the analytical criterion 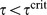, after less than one hour of biological time, and therefore puts a severe stability constraint on 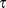. Moreover the theory is able to quantitatively confirm the timescale 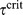 emerging from the spiking network simulations and allows us to see the detailed parameter dependence. In particular for a background rate of 3 Hz and the learning rate 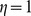 we find a critical timescale of 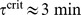 (simulations: 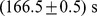, mean field model: 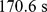).

In summary, our mean field model discussed here makes accurate quantitative predictions about the stability of a large spiking network model with plastic synapses for a given timescale of homeostasis. Furthermore it gives useful insights into parameter dependencies which are computationally costly to obtain from parameter sweeps in simulations of spiking networks. Our theory confirms that metaplastic triplet STDP with biological learning rates has to be matched by a homeostatic mechanism that acts on a timescale of seconds to minutes. In the next sections we will show that the mean field framework described here can be readily extended to other forms of homeostasis.

### Weight decay

**Figure 5:**
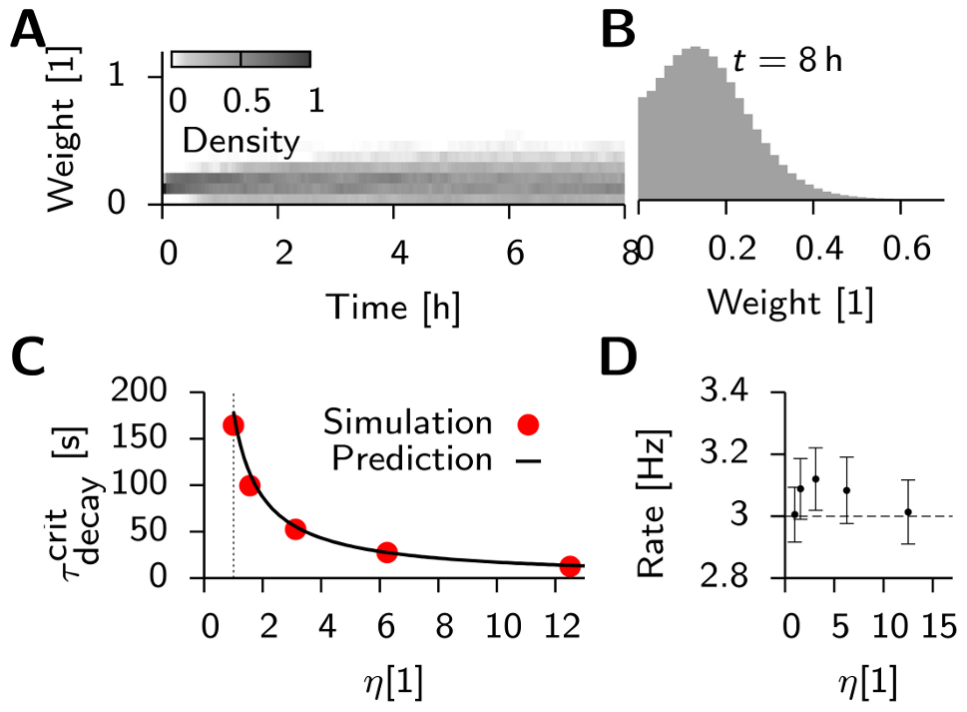
Slow synaptic weight decay renders weight distribution unimodal, but hardly affects global stability. (**A**) Evolution of the synaptic weight distribution over 8 h of background activity. (**B**) Synaptic weight distribution at 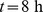. (**C**) Predictions for 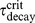
of mean field theory (solid line) and values obtained from direct simulation (points). (**D**) Final population firing rate as a function of 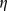 for values of 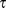 where the background state is a stable fixed point (dashed line: target rate 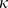; error bars: standard deviation over 100 bins of 1 s).

The induction of synaptic plasticity is only a first step towards the formation of long-term memory. In the absence of neuromodulators necessary to consolidate early LTP into late LTP, these modifications have been found to decay away with a time constant of 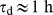 [40]. To study the effect of a slow synaptic decay on the stability of the background state we focus on the early phase of plasticity. In particular we neglect consolidation in the model and introduce a slow decay term
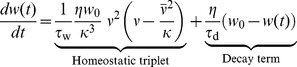
(8)where we already replaced the STDP rule by its equivalent rate based rule (see [30] and Methods, Eq. (17)), while the effect of the decay term can be written identically in the rate based model and the STDP model. Note that for 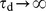 we retrieve the model studied in Figures 1–4. Again we determine the critical timescale of homeostasis in numerical simulations of the spiking network by systematically varying 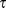 for different values of 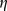. We further find that the slow weight decay causes the synaptic weights to stabilize in a unimodal distribution (Figure 5 A and B) which is fundamentally different to what we observed for the decay-free case. However, the critical time constant of homeostasis 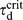 is only marginally larger than in the decay-free case (Figure 5 C).

To assess the impact of the decay on the critical timescale, the mean field approach, as it was derived above, can be adapted to take into account the constant synaptic decay (Methods). Provided the decay time constant is sufficiently long, we find the critical time constant to be
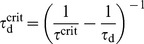
(9)which is in good agreement with the results from direct simulations (Figure 5 C). From Eq. (9) we can further confirm that the decay term only causes a small positive shift in the critical time constant as it was also observed in the spiking network. Furthermore, we see that the population firing rate settles to values closer to the actual target rate 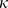 (Figure 5 D) than this was the case in the decay-free scenario.

In summary, adding a slow synaptic weight decay to the plasticity model is sufficient to cause substantial change to the steady state weight distribution in the network. Nevertheless this slow process does not affect the need for a rapid homeostatic mechanism.

### Synaptic scaling

**Figure 6:**
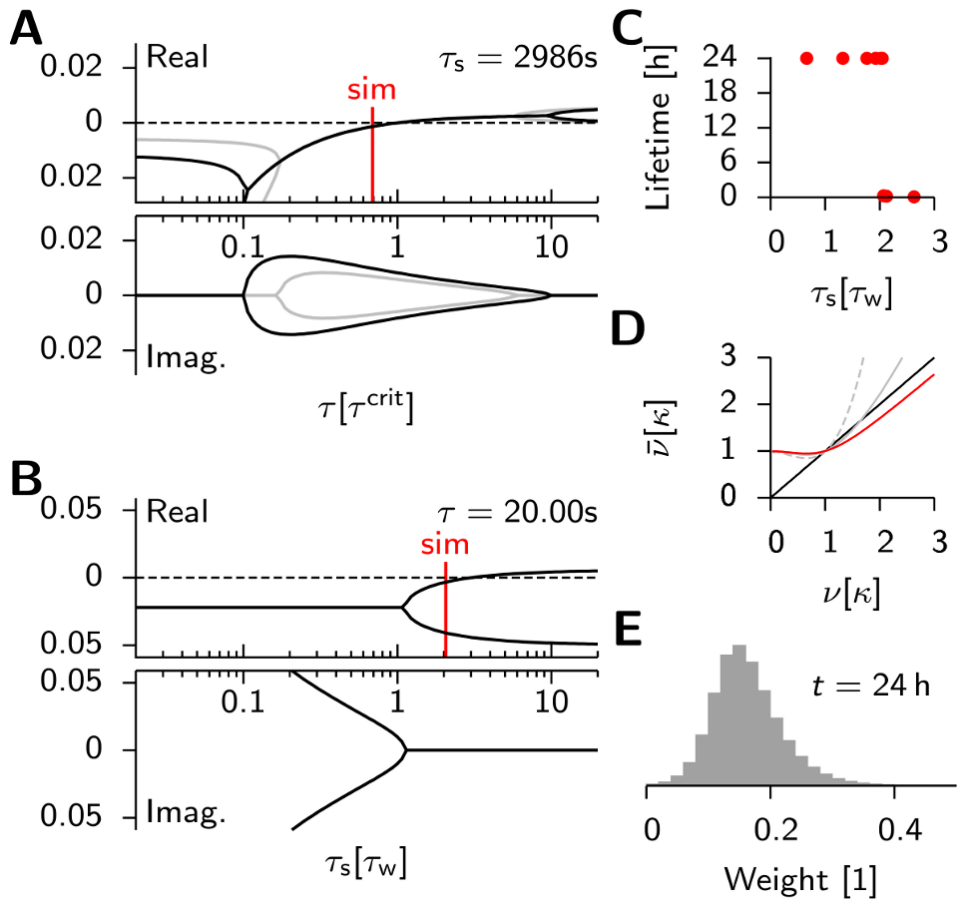
Triplet STDP with synaptic scaling requires a fast rate detector. (**A**) Black line: Eigenvalues of the Jacobian (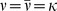) for different values of 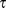 (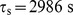). Gray curve: Values from Figure 3 B for reference. The red line (“sim”) indicates the critical value as obtained from simulating the full spiking network. (**B**) As before, but for different values of 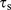 (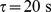). (**C**) Lifetimes of the background state in simulated networks of spiking neurons for different values of 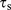 (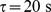). (**D**) Phase plane with nullclines. 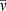-nullcline in black; 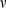-nullclines: dashed (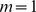), gray (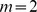) and red (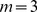). The latter was used in the rest of the figure. (**E**) Synaptic weight distribution after 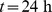 of simulation.

To test whether the previous findings are limited to our particular choice of metaplastic homeostatic mechanism, or whether they are also meaningful in the case of synaptic scaling [21] we now adapt the model by van Rossum et al. [35] and combine it with triplet STDP
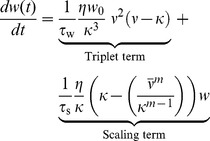
(10)where the rate of LTD is fixed in the triplet term (cf. Eq. (17)) and synaptic scaling is the only form of homeostasis. One important difference to the previous metaplastic STDP model is the addition of the scaling time constant 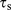 which controls the timescale of synaptic scaling. In the metaplastic model we analyzed above, this time constant is implicit since it is the same as the one of plasticity (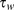). In contrast to the original model of synaptic scaling (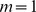 [35]) here we choose 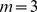 to avoid additional unstable fixed points in the phase plane (Figure 6 D).

Bearing this in mind we move on to linearizing the system around the fixed point of background activity (Methods). We find that for 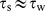 the eigenvalues of the linearized system qualitatively have the same shape as for the plasticity rule with homeostatically modulated LTD (Figure 6 A). In fact for sensible values of 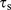, the stability condition is exactly the same: 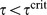 (cf. Eq. (7)). However, in the case of synaptic scaling Eq. (7) represents a necessary, but not a sufficient condition for stability. For too large values of 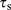 stability is lost also in the case of 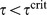 (Figure 6 B). On the other hand decreasing 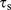 indefinitely leads to oscillations without any further effect on stability (see Methods and [35]).

To compare these findings with the equivalent STDP rule we perform numerical simulations with the full spiking network in which we set 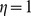 and choose 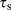 on the order of 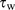 (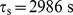). By changing 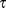 systematically (Figure 6 C) we determine the critical value to be smaller than predicted (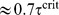), but within the same order of magnitude (Figure 6 A,C). Conversely when we start with 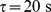 held fixed, we determine the critical value of 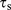 to be on the same order as 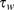 (Figure 6 B). At the end of a stable simulation run (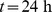) we find that synaptic weights have formed a unimodal distribution (Figure 6 E), an expected behavior of synaptic scaling [35].

In summary we have shown here that a fast rate detector is necessary to produce fast homeostatic responses to guarantee stable network dynamics also for the case of synaptic scaling. Although the quantitative agreement between the mean field model and the full spiking simulation is less accurate than in the case of for the metaplastic model above, both models confirm that the rate detector has to act on a timescale of seconds to minutes. Furthermore the time constant of the scaling term 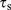 has to be comparable to the time scale of plasticity (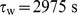) or stability is compromised, when 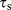 is chosen too large (and oscillations occur, when chosen too small).

## Discussion

In this paper we have shown that a realistic additive triplet STDP rule [30] can sustain a stable background state in balanced networks provided there is a homeostatic mechanism with a fast rate detector that acts on a timescale of seconds to minutes. We confirmed this result in a generic two dimensional mean field model in which the stability of the background state is interpreted as the linear stability of a non-zero fixed point of the system for which the timescale of the homeostatic rate detector 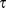 plays the role of a bifurcation parameter. These results are generic, i.e. independent of model details. In particular, we showed that similar results are obtained for triplet STDP with a form of metaplastic homeostasis, where homeostasis was implemented as a modulation of the LTD rate, or alternatively in combination with synaptic scaling. The mean field formalism produces accurate quantitative predictions for metaplastic triplet STDP. Although, in the case of triplet STDP in combination with synaptic scaling, the match of mean field model and direct simulations was less accurate, both support the notion that a fast rate detector is required for stability. For the case of synaptic scaling we found additionally that the homeostatic changes have to be implemented on a timescale comparable to the one of plasticity itself (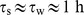), which is fast compared to most homeostatic mechanisms reported in the experimental literature, but consistent with earlier simulation studies that used fast homeostasis [13], [16]–[19], [35].

### Homeostasis and plasticity

The fact that Hebbian learning has to be opposed by some kind of compensatory mechanism has long been known [13]–[16] and such mechanisms indeed have been found [20], [36], [41]. In the following we will briefly review the different types of homeostasis affecting synaptic weights and how they relate to what was used in the present study.

Homeostasis can be classified in two main categories. We call models “weight homeostasis” if they try to keep all afferent weights into a cell normalized [13]. Such models have been criticized because they are non-local [15], i.e. they require cell wide spatial averaging over synapses, which can only be achieved in a plausible way if all synaptic weights decay at a global rate modulated by the total afferent synaptic strength [16]. To avoid this, “rate homeostasis” models have been proposed [15] which strive to maintain a certain postsynaptic firing rate. This approach, which we chose in the present study, has more experimental support [28], [29]. In contrast to the spatial filtering as described above, this mechanism requires temporal filtering of the postsynaptic rate over a given time window (represented by 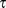 in this study). We can further distinguish between two principal types of homeostasis. A homeostatic mechanism can either act on the synaptic weights directly (e.g. synaptic scaling), or indirectly through metaplasticity [20], by changing parameters of the plasticity model over time. The former, direct form of homeostasis allows for synaptic changes even in the absence of activity as it is seen in synaptic scaling experiments [21] on a timescale of days. This is in contrast to theoretical models that apply scaling by algorithmically enforcing weight normalization [13], [18] on the timescale of one or a few simulation time-steps.

**Figure 7:**
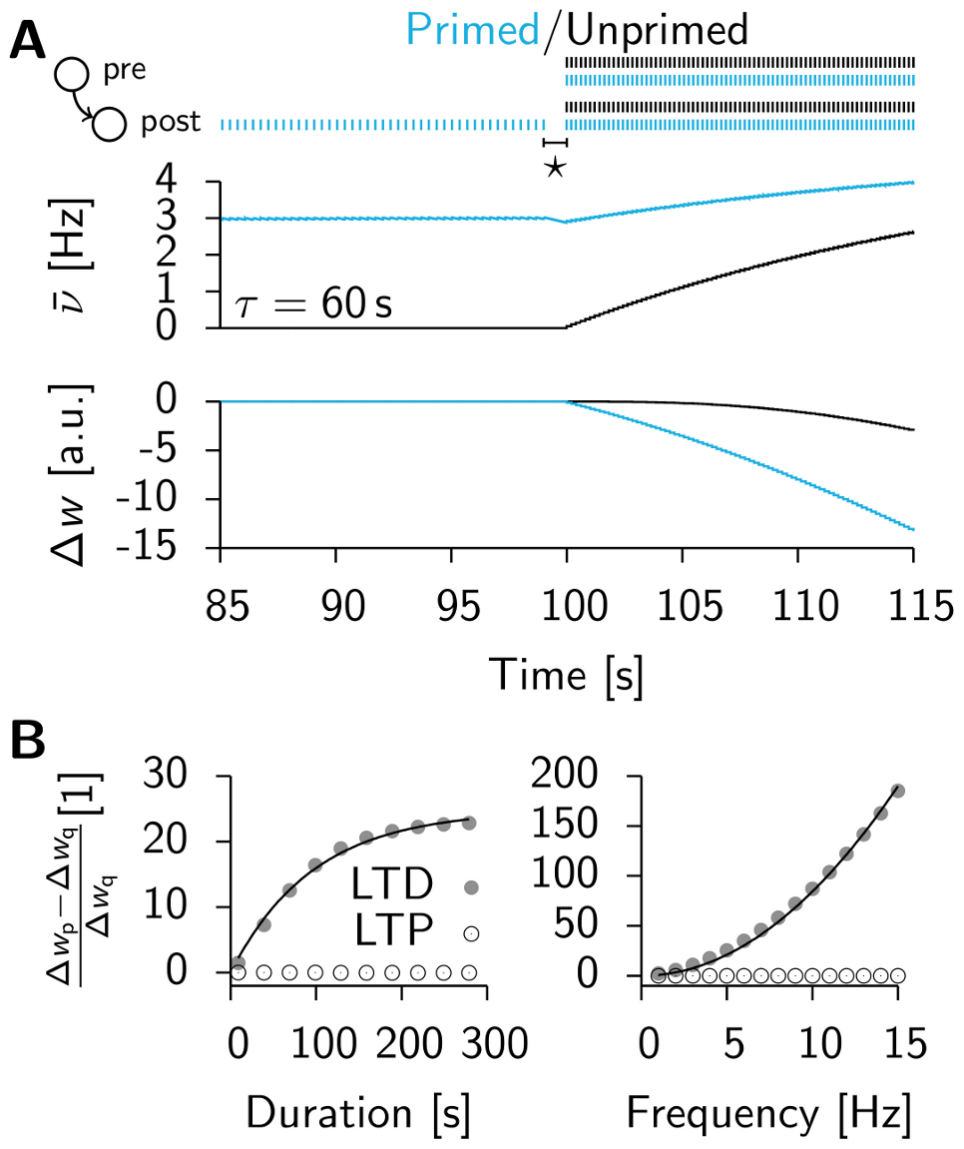
Postsynaptic priming affects STDP protocols. Simulation of the metaplastic triplet STDP rule [30]. (**A**) Top: Typical protocol for the induction of LTD (75 pairs (post-pre) at 5 Hz with −10 ms spike offset) in the triplet STDP model (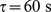) with a postsynaptic cell which is quiescent prior to the LTD protocol (black) compared to induction after postsynaptic priming (blue). Top, left: Pre- and postsynaptic spikes for priming and. Top, right: LTD induction. Middle: postsynaptic rate estimate 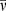 of the postsynaptic cell. Bottom: Weight change 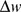 over time. Postsynaptic priming period (duration 100 s): regular firing at 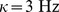 terminated by one second of silence (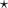) to avoid triplet effects. (**B**) Relative differences in final weight change between quiet (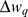) and primed protocol (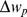) at the end of a LTD (gray) plasticity protocol. LTP protocol for reference (hollow, same paring protocol, with reversed timing, +10 ms spike offset). Left: For different durations of the priming period and fixed priming frequency of 3 Hz. Right: Different priming frequencies with fixed priming duration of 60 s. The black line is a RMS fit to LTD data points of: (left) an exponential function; (right) of a quadratic function.

In our study we looked at both approaches. In the metaplastic triplet STDP model homeostasis manifest itself as a shift in the plasticity threshold between LTD and LTP [19], [30], [42], [43]. This is achieved by modulating the rate of LTD induction using the temporal average of the postsynaptic firing rates over a given time window (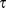). As we have shown, this average has to follow the neuronal spiking activity very rapidly, meaning that plasticity parameters change on a short timescale, which is comparable to the duration of many standard STDP protocols [26]. We therefore predict that if biological circuits rely on such a metaplastic homeostatic mechanism, weight changes are different for cells that are silent prior to a plasticity induction than for cells that have been primed by postsynaptic firing (over an extended period before the induction protocol). In Figure 7 A we demonstrate this idea in the model of metaplastic triplet STDP (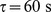) for a typical LTD induction protocol (75 pairs at 5 Hz with −10 ms spike offset). Figure 7 B shows the relative differences between primed and unprimed experiments in dependence of the length of the priming duration or the priming frequency respectively. Since this plasticity rule implements homeostasis as an activity dependent change of the LTD learning rate, the amount of LTD changes dramatically while LTP is unaffected by priming. However, we expect that the main results of our mean field analysis also hold for cases in which LTP is affected, as long as the net synaptic weight change decreases with the intensity of priming. In either case the functional form of the dependence allows us to draw conclusions on the order of magnitude of 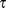 and the exponent of 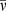 appearing in 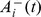 (cf. Eq. 18). Conversely, if homeostasis was exclusively mediated by synaptic scaling, we would expect that it manifests as a heterosynaptic effect. Its impact, however, would likely be smaller than in the case of metaplastic triplet STDP, because synaptic scaling does not have an explicit dependence on the presynaptic firing rate.

Since stability requires 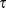 to be relatively short, it is also worth considering the extreme case where it is on the timescale of a few hundred milliseconds. In that case the learning rule can be interpreted as a quadruplet STDP rule combining a triplet term for LTP (e.g. post-pre-post) with a quadruplet term for LTD (e.g. post-post-post-pre). While such a choice of 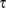 would make sense from a stability point of view, this behavior is not seen in experiments [26].

### Influence of the model design

The timescales of synaptic plasticity and the time constants behind most homeostatic mechanisms reported in experiments are far apart. While plasticity can cause substantial synaptic changes in less than one minute [24]–[26], homeostatic responses typically differ on the order of several magnitudes (hours or days) [29], [37]. In this paper we have shown that even if homeostatic changes manifest relatively slowly they have to be controlled by a fast rate detector, else triplet STDP is incompatible with the low background activity observed in cortical circuits. We argue that this statement is likely not to be limited to our particular model, but rather applies to an entire family of existing plasticity models.

The basic building blocks of our study were a network model and a homeostatic plasticity rule. We used a generic balanced network model [7], [10], [44]–[46] to mimic brain-like spiking activity in a recurrent neural network. It is clear that the particular choice of network model does affect our results in a quantitative way and absolute predictions would require a more accurate and detailed network model. Nevertheless, we expect homeostasis to have similar timescale requirements in more detailed models as well. Indeed, as long as a strengthening of the excitatory synapses yields increased firing rates without a major change in the correlations, the qualitative predictions of the mean field model hold. However, our simulations were limited to roughly 1000 recurrent inputs per neuron, which is presumably less than what real cortical neurons receive [31], so that excitatory run-away could build up even more rapidly in real networks than in our simulations.

The second building block of our model was the plasticity rule. Here we chose triplet STDP [30] as a plasticity model that quantitatively captures a large body of experiments [24], [26]. One key feature of this model, which is seen across a range of in-vitro plasticity studies, is the fact that it yields LTP for high postsynaptic firing rates. The emergence of a critical timescale for homeostasis is mainly rooted in this fact and it is largely relaxed for pair-based STDP, be it additive or multiplicative [12]. However, such models do not capture experimental data as well as triplet STDP.

With the models we analyzed, namely the metaplastic triplet STDP and triplet STDP with synaptic scaling, we combined a realistic STDP learning rule with two quite different, but commonly used synaptic homeostatic mechanisms [15], [18], [19], [30], [35], [38], [42], [43], [47], [48]. The fact that we were able to show in both cases, either using a generic mean field model or numerical simulations of large balanced networks, that a fast rate detector is needed for stability, suggests that these results are quite general. The argument is further strengthened by the fact that existing computational models demonstrating stable background activity in plastic recurrent network models either use a form of multiplicative STDP which can be intrinsically stable [12], but has poor memory retention [12], [34], or rely on a fast homeostatic mechanism [18], [43]. In fact one of the first studies that illustrates stable learning in large recurrent networks combined with long memory retention times [43] is a model of metaplasticity built on top of the triplet model [30]. To describe effects observed in priming experiments [41], [49], [50], the authors introduce two floating plasticity thresholds that modulate the rate of LTP and LTD depending on the low-pass filtered neuronal activity. El Boustani and colleagues obtain the time constants behind these filters by fitting their model to experimental data. It is striking, and in agreement with what we report here, that the timescales they find are on the order of 1 s [43].

We conclude that current plasticity models that capture experimental data well require homeostasis to be able to react fast in order to maintain a stable background state. Likewise, if there is no rapid homeostatic control, most current plasticity models are probably missing a key ingredient to what makes cortical circuits stable.

### Experimental evidence

The metaplastic triplet STDP rule we used makes use of an homeostatically modulated rate of LTD and can be mapped to a BCM-like learning rule [30], [38]. The BCM theory relies on a plasticity rule with a neuron wide sliding threshold [15], [51]. There seems to be some experimental ground for this idea [52], [53] and it is intriguing, that the effects reported there are on the order of 30 min or less which points towards a relatively fast mechanism. We should further point out, that the arguments that led us to the critical timescale of homeostasis are not limited to a neuron wide sliding threshold. In fact the mean field equations for a global or local synaptic sliding threshold, or even one based on local dendritic compartments, are identical. Therefore the arguments we put forward also hold for the latter cases, which have experimental support through priming experiments [41], [49], [50]. Priming experiments highlight changes in the induction of plasticity which depends on the synaptic activity over some 30 min.

With synaptic scaling we studied another possibility of introducing homeostasis into the triplet STDP model. Homeostatic scaling of synapses has good experimental support [21], [29], [37]. Although it is generally associated with long timescales (order of days), also more rapid forms of scaling are known [54]–[56] of which some indeed act on the order of minutes [57]. Further modeling is required to test the ability of these rapid forms of homeostasis to guarantee stability in recurrent networks.

Finally one should note that the critical time scale of the rate detector strongly depends on the firing rates of the background state (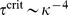, cf. Eq. (7) and Methods). The low firing rates reported experimentally [58]–[60] are therefore potentially necessary to guarantee the stability of the network. Conversely, cells or sub-networks with higher mean firing rates should have lower learning rates in order to be stable.

### Limitations

Despite the mean field formalism being a drastic simplification of the original spiking model, the results we were able to derive from it were surprisingly accurate in the case of metaplastic triplet STDP and off by a factor of two in the case of triplet STDP with synaptic scaling. In all cases our mean field predictions overestimate the critical timescale obtained from simulations. This discrepancy has multiple potential reasons. First, in the mean field model we completely omit the existence of noise, fluctuations, and correlations. That these factors do play a role follows from the observation that the spiking network does not stabilize at the target rate 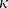, but at higher values (cf. Figure 2 E). Although correlations in the AI state are small, they are on average positive [9]. When we estimated 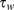 we explicitly ignored correlations and required that LTD and LTP cancel at a firing rate 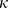. Adding correlations causes this cancellation to take place at slightly higher rates, which reduces the effective critical time constant. In the rate formulation of the STDP rule we make the simplifying assumption that the synaptic traces are perfect estimates of the postsynaptic firing rates. Indeed it can be shown that fluctuations that are present in the rates, bias the learning rule towards LTP (see Text S1). Finally, any deviation of the population activity from its target value, initial or spontaneous, can be thought of as perturbations around the fixed point of background activity in the mean field model. This can compromise stability when the basin of attraction is small, as is the case when 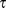 is close to criticality (Figure 3 D). Again, such perturbations bias the critical value for the spiking network towards lower values. All the above points concern the simplifications made when going from the spiking model to the mean field model.

More importantly, the spiking model itself already represents a drastic simplification of the biological reality. For instance, we did not include neuronal firing rate adaptation or synaptic short-term plasticity (STP) in the present model. The timescales involved in firing rate adaptation are typically short (on the order of 100 ms) and their effect therefore negligible at the low firing rates of background activity [61], [62]. While the time constants behind STP can be longer than that, their stabilizing effect is somewhat less clear since they can be facilitating and depressing [63]. Although we do not expect STP to have a strong impact on our main results, it would be an interesting avenue to verify this in future studies.

All our present studies were limited to spontaneous background activity. In a more realistic scenario we would expect the network to receive external input with spatio-temporal correlations. Such input will generally cause synaptic weights to change, which in the mean field model corresponds to a perturbation of the dynamical network state around the stable fixed point. If the perturbation leaves the system in the basin of attraction of background activity, equilibrium will be restored over time. If, however, the perturbation is strong, or perturbations are in rapid concession and start to pile up, the system loses stability once its dynamical state reaches the separatrix (cf. Figure 3 C,D).

Another possibility worth mentioning is homeostatic regulation through inhibitory synaptic plasticity (ISP) [64]–[68]. Recent theoretical studies [69]–[71] suggest that ISP could produce an intrinsically stable feed-back system. Although we cannot exclude ISP as an important factor in network homeostasis, we have excluded it in the current study. It is likely that to stabilize Hebbian plasticity at excitatory synapses, ISP has to act on a comparable timescale [72] and it will be interesting to integrate future experimental findings into a similar framework as presented here.

### Conclusion

In summary, homeostatic mechanisms are necessary to stabilize the background activity in network models subject to Hebbian plasticity. Homeostasis needs to react faster than what is experimentally observed. This raises the important question of how the background activity in the brain can be stable. Our results suggest that the existence of a rapid homeostatic mechanism could be one possible answer. That, however, would require this mechanism to act on the same timescale as most STDP induction protocols. This then raises the question, why it has not been observed so far. Suitable plasticity protocols to detect such a mechanism should be similar to priming experiments [41], [49], but on the timescale of 1 min (Figure 7). Another possibility would be, that the plasticity rate 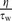 is not a constant after all, but subject to some neuromodulatory change [73]. This could be possible, since it cannot be excluded that conditions in slice preparations, like the ones used to obtain the parameters of triplet STDP [26], are different from in-vivo conditions. Finally, also fast forms of ISP could play a role in network stability.

No matter whether through ISP or additional, hitherto unseen excitatory homeostatic effects, a variation of current models of homeostasis and plasticity seem inevitable, to achieve stability in plastic network models whilst making them biologically plausible.

## Methods

To study stability in plastic spiking recurrent networks we simulated networks of 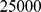 integrate-and-fire neurons with conductance-based synapses (Figure 1 A). The size of the network was chosen large enough to allow for an asynchronous irregular (AI) background state with low spiking correlations, but still small enough to enable simulations over long periods of biological time.

### Neuron model

The networks we study consist of leaky integrate-and-fire neurons with a relative refractory mechanism connected by conductance-based synapses [46]. The membrane voltage 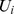 of neuron 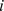 evolves according to
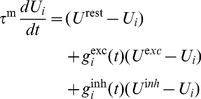
(11)A spike is triggered when 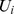 crosses the spiking threshold 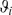. After a spike 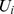 is reset to 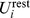 and the threshold 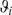 is increased 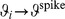 to implement refractoriness. In the absence of spikes the threshold relaxes back to its resting value 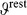 according to
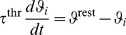
(12)with 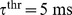 similar to [42]. Inhibitory neurons were modeled identically except for a shorter membrane time constant 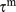. All relevant parameters are summarized in Table 1.

**Table 1:** Neuron model and synaptic parameters.

Membrane		Threshold		Synapse	
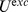	0 mV	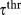	5 ms	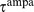	5 ms
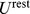	−70 mV	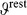	−50 mV	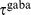	10 ms
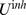	−80 mV	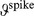	100 mV	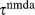	100 ms
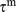	20 ms (10 ms*)			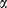	0.5

*) only inhibitory neurons.

The spike train 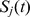 of neuron 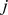 is defined as 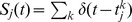, where the sum runs over all 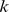 corresponding firing times 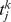 of neuron 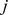. It affects the synaptic conductances of downstream neurons as
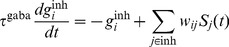
(13)if the index 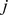 corresponds to an inhibitory neuron or
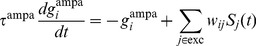
(14)

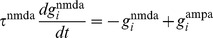
(15)in the case of an excitatory cell. Here 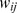 is the weight of the synapse connecting neuron 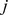 with 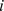 (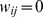 if the connection does not exists). Excitatory synapses contain a fast rising AMPA component with exponential decay and a slowly rising NMDA component with its respective exponential decay with time constant 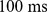. For simplicity we implemented the NMDA component as a low pass filtered version of the AMPA conductance (Eq. (15)). The complete excitatory postsynaptic potential (EPSP) is then given by a weighted sum of the AMPA and NMDA conductances
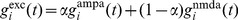
(16)With the chosen parameters (cf. Table 1), a typical EPSP has an amplitude of about 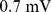, as shown in Figure 1 B. For computational efficiency the voltage dependence of NMDA channels was omitted.

### Network model

All units (20000 excitatory and 5000 inhibitory units, see Table 2 for details) are connected randomly with a sparse connectivity of 5%. Additionally each excitatory cell receives external input from a pool of 2500 independent Poisson processes firing at 2 Hz that are connected with 5% probability. The relevant synaptic weight values are summarized in Table 2. Due to the high recurrence (on average 1000 out of 1125 connections are from within the network) the mean firing rate and network activity are sensitive to small changes in the recurrent synaptic strength. By appropriate choice of the excitatory weights (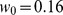) the network is initially tuned to the balanced state with AI activity at a mean population activity of approximately 3 Hz.

**Table 2:** Network model parameters.

Neuron groups and connectivity		Synaptic weight structure	
Neural population	Size	Connection	Weight
Excitatory (E)	20000	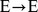	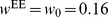
Inhibitory (I)	5000	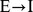	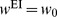
External Poisson (ext)	2500 at 2 Hz	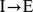	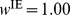
Network connectivity	5%	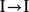	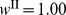
Connectivity from ext	5%	ext Poisson 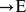	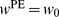

### Plasticity model

We model synaptic plasticity after the triplet STDP model of [30], using the minimal parameter set corresponding to in-vitro visual cortex data [26]. Plasticity only affects the EE recurrent connections. Weight updates 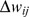 act additively on the matrix elements 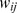 and are given by
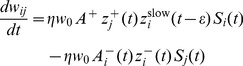
(17)where 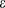 is a small positive number and 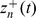, 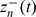 and 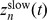 are synaptic traces of neuron 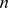 defined as 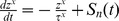 with associated time constants 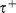, 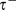 and 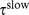 respectively (see Table 3 and [30]). Since the original triplet model describes relative synaptic changes, weight updates in Eq. (17) are scaled by the factor 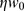, where 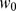 is the initial synaptic weight and 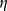 is an additional parameter that can be interpreted as a learning rate, or a conversion factor between the weight scales of the model and the true biological scale. In the model we approximate the biological scale by choosing plausible values for 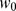 (cf. Figure 1 B) and therefore expect 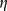 to be of the order of one. For a synapse with an initial weight of 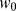, a value of 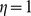 corresponds to the learning rate that best fits visual cortex data [30]. However, since small values of 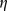 are computationally expensive we used 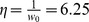 in Figure 2 to ensure that a stable weight distribution can be observed within a day of simulated biological time (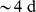 of computation time). Note that for 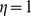 we would expect a comparable degree of convergence after 6.25 days of simulated time (roughly four weeks of computation). During ongoing plasticity the allowed weight values are limited to the interval 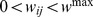. Note that to avoid the creation of new synapses, connections that have zero weight initially, remain absent (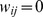) throughout the entire simulation.

**Table 3:** Plasticity model parameters.

Plasticity window	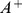	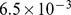
	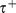	16.8 ms
	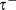	33.7 ms
	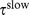	114 ms
Initial weight	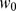	0.16
Weight limits	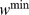	0
	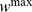	1
Target firing rate	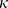	3 Hz
Rel. learning rate	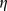	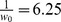 *
	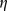	1 (Figure S1)

*) As used in Figures 2 and 4 B,C.

In simulations with metaplastic triplet STDP the amount of long term synaptic depression (LTD) 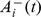 is varied homeostatically as a function of the moving average 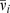 of the postsynaptic firing rate [15], [19], [30], [38] with
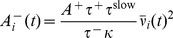
(18)This choice of 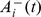 ensures that for uncorrelated Poisson firing at the rate 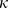 LTP and LTD cancel on average. The moving average 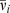 of the firing rate of neuron 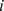 is implemented as a low pass filtered version of its spike train
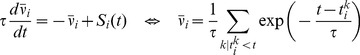
(19)where 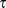 is the timescale which controls of the temporal evolution of 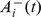 (cf. Eq. (18)).

In simulations that require an additional slow weight decay of the weights we approximate this exponential decay, to avoid the costly operation of updating all weights after each time step, by periodically (period 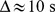) multiplying all weights by the factor 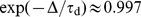. Finally, simulations of synaptic scaling are performed using a fixed value 
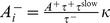
. The scaling of the weights is approximated with the same approach as for weight decay. In such cases 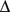 is adapted appropriately according to the occurring scaling time constant 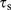.

### The time constant of plasticity

We determine the timescale of plasticity in the mean field model by approximating 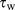 from the plasticity parameters of the triplet STDP model [30]. To do so we consider the expectation value of the mean weight update averaged over many spike pairs, and we assume that pre- and postsynaptic firing is uncorrelated with stationary rates 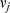 and 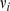 respectively. The average relative weight change over time then reads
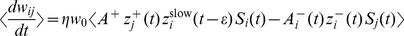
(20)

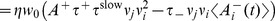
(21)

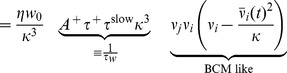
(22)The resulting differential equation can be directly identified with Eq. (4) to obtain the effective time constant 
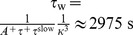
.

### Numerical simulations

All differential equations were integrated using forward Euler integration with a 0.1 ms time step. Spiking simulations were written in C++ using Open MPI and the Boost libraries. The sources were compiled using the GNU C compiler. Simulations were run on 5 Linux workstations equipped with Intel(R) Core(TM)2 Duo E8400 CPUs and 24 GB of RAM each. It took approximately four and a half days to simulate one day of biological time.

Numerical results for the phase plane analysis, such as the position of the separatrix, were obtained by integrating the ODEs of the mean field model numerically using custom-written Python code.

### Derivation of the stability condition in the mean field model

To analyze the stability of the fixed point of background activity (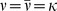) in the case of the metaplastic triplet STDP rule, we consider the Jacobian 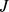 of the two dimensional system (cf. Eqs. (5),(6)) in the general case of 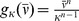 for 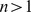.
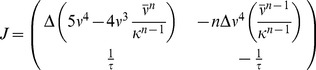
(23)where we introduced the auxiliary variable 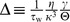. When evaluated at the fixed point 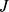 reduces to
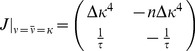
(24)with characteristic polynomial
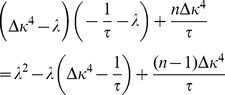
(25)which determines the eigenvalues to be of the linearized system at the fixed point of background activity
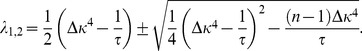
(26)


Stability of the fixed point requires all eigenvalues to have negative real parts (e.g. [74]). We now prove that the real part of both eigenvalues is negative if and only if 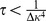. The square root in Eq. (26) is either purely imaginary, in which case 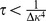 follows directly. For the case in which the square root is real we can express the larger of the two eigenvalues as
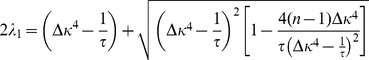
(27)

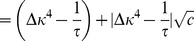
(28)where we introduced the variable 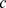 for the term in the square brackets (Eq. (27)). If 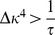 then 
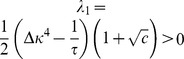
 and the fixed point is unstable. If, however, 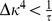 then we know
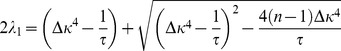
(29)

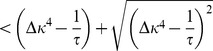
(30)

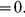
(31)Here, we used the fact that all occurring constants are positive, 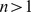 and the argument in the square root is positive as well. Finally we can conclude the fixed point is stable if 
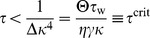
. This identifies 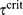 as an important limiting case for the stability of the fixed point. It is interesting to note that 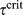 is independent of 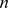.

#### Stability condition for weight decay

If we are to include an additional weight decay in the above model we replace Eq. (6) by
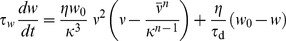
(32)and proceed similarly as before by replacing all occurrences of 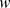. In the decay term we can use the identities 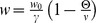 and since 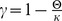 (cf. Eq. (3)) to rewrite
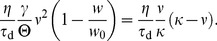
(33)We use this expression together with our results from Eq. (23) and the abbreviation 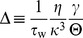, to arrive at
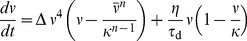
(34)which leads to the following Jacobian at the fixed point
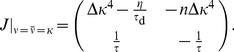
(35)The corresponding eigenvalues are given by
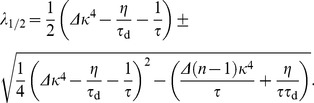
(36)


As we have seen earlier the stability is determined by the first term since the square root is purely imaginary around criticality. This leads us to the relaxed stability condition 
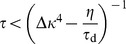
 and therefore with 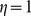 and 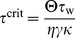 we get 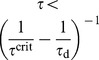.

#### Stability condition for synaptic scaling

Here we will derive the critical time constant 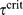 for yet another variation of the triplet rule
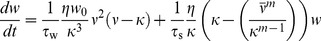
(37)which uses synaptic scaling to achieve the target rate 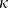 (cf. [35]). With the same transformations as before (i.e. 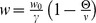) we can bring Eq. (37) to the form
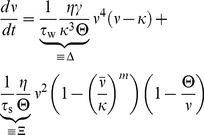
(38)which taken together with Eq. (5) yields the following Jacobian at the fixed point
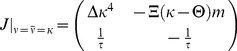
(39)with associated eigenvalues
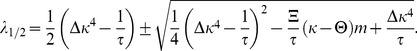
(40)


We can appreciate directly from Eq. (40) that the real part of the largest eigenvalue is lower bounded 
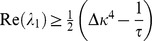
 and therefore we find that stability requires 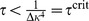, which is the same condition as above for the case of metaplastic triplet STDP. However, in the case of synaptic scaling this stability condition is necessary, but not sufficient. This we can see in Eq. (40) for given 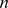, when 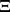 becomes sufficiently small (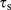 sufficiently large) eventually we get 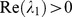, where the background state loses stability (cf. Figure 6 B). Hence, in addition to 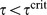 there is also a critical value for 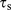 which can be on a comparable scale like 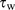, but not arbitrarily large.

## Supporting Information

Figure S1
**Evolution of the population rate for metaplastic triplet STDP model.** (**A**) Temporal evolution of mean population rate for different values of 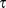 (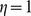). While the change in stability in the vicinity of 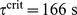 can be understood from the mean field theory, which also predicts the observed oscillations at criticality, the late destabilization of the curve 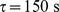 is not captured by the theory. (**B**) Evolution of mean population rate for 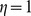. Black: 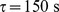 and weights are initialized with the weights from a stable run (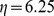, 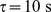) at 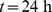. Cyan: Same, but with 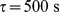. Dark blue: 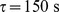, weight initialization as in (A), but maximally allowed weights limited to 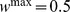. Light blue: 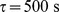, network falls silent at 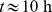. Purple: 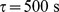, with 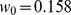 (the learning rate was unchanged), which reduces the initial excursion to low rates.(PDF)

Text S1 **Rate fluctuations.** Mean field solutions ignore the effect of fluctuations in the postsynaptic firing rate.
(PDF)
