# Supplementary material for: Synaptic Plasticity in Neural Networks Needs Homeostasis with a Fast Rate Detector
Source: PLoS Comput Biol. 2013 Nov 14;9(11):e1003330. doi: 10.1371/journal.pcbi.1003330 (PMC3828150; doi:10.1371/journal.pcbi.1003330)
Supplement: Figure S1 — Evolution of the population rate for metaplastic triplet STDP model. (A) Temporal evolution of mean population rate for different values of (). While the change in stability in the vicinity of can be understood from the mean field theory, which also predicts the observed oscillations at criticality, the late destabilization of the curve is not captured by the theory. (B) Evolution of mean population rate for . Black: and weights are initialized with the weights from a stable run (, ) at . Cyan: Same, but with . Dark blue: , weight initialization as in (A), but maximally allowed weights limited to . Light blue: , network falls silent at . Purple: , with (the learning rate was unchanged), which reduces the initial excursion to low rates. (PDF) [file pcbi.1003330.s001.pdf]

Figure S1

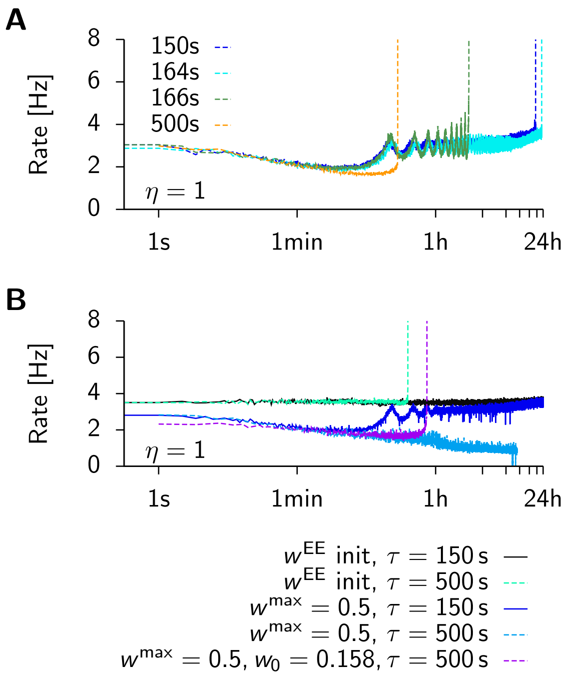

Evolution of the population rate for metaplastic triplet STDP model. (**A**) Temporal evolution of mean population rate for different values of τ ($\eta = 1$). While the change in stability in the vicinity of $\tau^{\mathrm{crit}} = 166\,\mathrm{s}$ can be understood from the mean field theory, which also predicts the observed oscillations at criticality, the late destabilization of the curve $\tau = 150\,\mathrm{s}$ is not captured by the theory. (**B**) Evolution of mean population rate for $\eta = 1$. Black: $\tau = 150\,\mathrm{s}$ and weights are initialized with the weights from a stable run ($\eta = 6.25$, $\tau = 10\,\mathrm{s}$) at $t = 24\,\mathrm{h}$. Cyan: Same, but with $\tau = 500\,\mathrm{s}$. Dark blue: $\tau = 150\,\mathrm{s}$, weight initialization as in (A), but maximally allowed weights limited to $w^{\mathrm{max}} = 0.5$. Light blue: $\tau = 500\,\mathrm{s}$, network falls silent at $t \approx 10\,\mathrm{h}$. Purple: $\tau = 500\,\mathrm{s}$, with $w_0 = 0.158$ (the learning rate was unchanged), which reduces the initial excursion to low rates.
