# Supplementary material for: Synaptic Plasticity in Neural Networks Needs Homeostasis with a Fast Rate Detector
Source: PLoS Comput Biol. 2013 Nov 14;9(11):e1003330. doi: 10.1371/journal.pcbi.1003330 (PMC3828150; doi:10.1371/journal.pcbi.1003330)
Supplement: Text S1 — Rate fluctuations. Mean field solutions ignore the effect of fluctuations in the postsynaptic firing rate. (PDF) [file pcbi.1003330.s002.pdf]

Text S1: Rate fluctuations

The approximation that κ is the fixed point of our plasticity rule is only true in a rate model where all rates take their respective mean value. Here we show that in the presence of rate fluctuations the actual fixed point is shifted to higher values. Since a neuron receives many inputs at any moment in time, we ignore fluctuations in the presynaptic rate, and focus on the postsynaptic side. First we analyze the effect on the synaptic weights of a single unit with fluctuating rate ν_i ($\left\langle \nu_i^2 \right\rangle = \nu^2 + \sigma^2$). We will furthermore neglect any fluctuations in $\bar{\nu}_i$. For the purely additive learning rule weights are stable on average if $\tau_w \left\langle \frac{dw}{dt} \right\rangle = 0$. Applying the temporal average to the rate based learning rule

$$\tau_w \frac{dw}{dt} \propto \nu \nu_i \left(\nu_i - \frac{\bar{\nu}_i^2}{\kappa} \right) \tag{41}$$

and requiring it to be equal to zero gives

$$\begin{aligned} \nu \left\langle \nu_i^2 \right\rangle - \frac{\nu^3}{\kappa} \left\langle \nu_i \right\rangle &= 0 &\quad (42) \\ \nu^2 + \sigma^2 - \frac{\nu^3}{\kappa} &= 0 &\quad (43) \\ \nu &= \kappa + \frac{\sigma^2}{\nu^2} &\quad (44) \end{aligned}$$

where we assumed $\nu > 0$. Therefore the stable rate ν is always higher than κ. This intuition breaks down if τ is chosen too short. For the case $\tau\nu < 1$ fluctuations in $\bar{\nu}_i$ become substantial.

By ignoring both correlations and fluctuations in the mean field model systematic errors are introduced that underestimate the actual resulting target rate.
